# Efficacy of pelvic floor muscle training and hypopressive exercises for treating pelvic organ prolapse in women: randomized controlled trial

**DOI:** 10.1590/S1516-31802012000100002

**Published:** 2012-02-16

**Authors:** Bruno Teixeira Bernardes, Ana Paula Magalhães Resende, Liliana Stüpp, Emerson Oliveira, Rodrigo Aquino Castro, Zsuzsanna Ilona Katalin Jármy di Bella, Manoel João Batista Castello Girão, Marair Gracio Ferreira Sartori

**Affiliations:** I MD. Physician and Assistant Professor, Department of Gynecology and Obstetrics, Universidade Federal de Uberlândia (UFU), Uberlândia, Minas Gerais, Brazil.; II Physiotherapist and Doctoral Student, Department of Gynecology, Universidade Federal de São Paulo (Unifesp), São Paulo, Brazil.; III MD, PhD. Physician, Department of Gynecology, Universidade Federal de São Paulo (Unifesp), São Paulo, Brazil.; IV MD, PhD. Physician and Adjunct Professor, Department of Gynecology, Universidade Federal de São Paulo (Unifesp), São Paulo, Brazil.; V MD, PhD. Physician and Affiliated Professor, Department of Gynecology, Universidade Federal de São Paulo (Unifesp), São Paulo, Brazil.; VI MD, PhD. Physician and Titular Professor, Department of Gynecology, Universidade Federal de São Paulo (Unifesp), São Paulo, Brazil.; VII MD, PhD. Physician and Associate Professor, Department of Gynecology, Universidade Federal de São Paulo (Unifesp), São Paulo, Brazil.

**Keywords:** Ultrasonography, Pelvic floor, Exercise, Perineum, Hypertrophy, Ultrassonografia, Soalho pélvico, Exercício, Períneo, Hipertrofia

## Abstract

**CONTEXT AND OBJECTIVE:**

Previous studies have shown that women with pelvic floor dysfunctions present decreased cross-sectional area (CSA) of the levator ani muscle. One way to assess the effects of training programs is to measure the CSA of the muscle, using ultrasonography. The aim here was to evaluate the efficacy of pelvic floor muscle training and hypopressive exercises for increasing the CSA of the levator ani muscle in women with pelvic organ prolapse.

**DESIGN AND SETTING:**

Prospective randomized controlled trial at the Urogynecology outpatient clinic of Universidade Federal de São Paulo.

**METHODS:**

Fifty-eight women with stage II pelvic organ prolapse were divided into three groups for physiotherapy: a pelvic floor muscle training group (GI); a hypopressive exercise group (GII); and a control group (GIII). The patients underwent transperineal ultrasonographic evaluation using a transducer of frequency 4-9 MHz. The (CSA) of the levator ani muscle was measured before physiotherapy and after 12 weeks of treatment.

**RESULTS:**

The groups were homogeneous regarding age, number of pregnancies, number of vaginal deliveries, body mass index and hormonal status. Statistically significant differences in CSA were found in GI and GII from before to after the treatment (P < 0.001), but not in relation to GIII (P = 0.816).

**CONCLUSIONS:**

The CSA of the levator ani muscle increased significantly with physiotherapy among the women with pelvic organ prolapse. Pelvic floor muscle training and hypopressive exercises produced similar improvements in the CSA of the levator ani muscle.

**CLINICAL TRIAL REGISTRATION NUMBER:**

NCT01196598

## RESUMO

**CONTEXTO E OBJETIVO:**

Estudos anteriores mostraram que mulheres com disfunção do assoalho pélvico possuem diminuição da área de secção transversal (AST) do músculo levantador do ânus. Uma forma de avaliar os efeitos de um programa de treinamento é mensurar a AST do músculo por ultrassonografia. Oobjetivo foi avaliar a eficácia do treinamento da musculatura do assoalho pélvico e de exercícios hipopressivos no aumento da AST do músculo levantador do ânus em mulheres com prolapso de órgãos pélvicos.

**TIPO DE ESTUDO E LOCAL:**

Estudo prospectivo, randomizado e controlado realizado no Ambulatório de Uroginecologia da Universidade Federal de São Paulo.

**MÉTODOS:**

Cinquenta e oito mulheres com prolapso de órgãos pélvicos estádio II foram divididas em três grupos para tratamento fisioterapêutico: (GI) grupo de treinamento dos músculos do assoalho pélvico, (GII) grupo de exercícios hipopressivos e (GIII) grupo controle. As pacientes se submeteram a avaliação ultrassonográfica transperineal com transdutor de frequência 4-9 MHz. Foi mensurada a AST do músculo levantador do ânus antes e após 12 semanas de tratamento fisioterapêutico.

**RESULTADOS:**

Os grupos foram homogêneos no que se refere a idade, número de gestações, número de partos vaginais, índice de massa corpórea e estado hormonal. Diferença significante foi observada na AST de GI e GII antes e após o tratamento (P < 0,001) e isso não ocorreu com o GIII (P = 0,816).

**CONCLUSÕES:**

A AST do músculo levantador do ânus aumentou significativamente com tratamento fisioterapêutico em mulheres com prolapso de órgãos pélvicos. Treinamento dos músculos do assoalho pélvico e exercícios hipopressivos são semelhantes no que se refere ao aumento da AST do músculo levantador do ânus.

## INTRODUCTION

Ultrasonography has been widely used to detect dysfunction in the pelvic floor muscles (PFM),^1^^,^^2^^,^^3^^,^^4^ to determine the effect of childbirth in these muscles and to evaluate the effectiveness of muscle training.^4^^,^^5^ Three-dimensional ultrasound is the most common type; however, two-dimensional ultrasound may be useful for evaluating the pelvic floor.^6^^,^^7^

Many randomized controlled trials and systematic reviews have shown that PFM training is effective for treating stress urinary incontinence, with success rates of between 44% and 80% among adult females.^8^^,^^9^ However, there is little evidence that it can improve pelvic organ prolapse and its symptoms.^10^

Some theories have been proposed in order to explain the mechanism of action of PFM training in pelvic organ prolapse. Behavioral therapy, which teaches women to contract the PFM before and during increases in abdominal pressure, helps prevent pelvic organ prolapse. Strength training to increase muscle volume raises the levator plate and decreases the genital hiatus, thereby promoting greater support for the pelvic organs.^10^^,^^11^

Previous studies have shown that women with pelvic organ prolapse present decreased cross-sectional area (csa) of the levator ani muscle, increased genital hiatus and decreased muscle strength.^12^^,^^13^ one way to assess the effect of muscle training is to measure the csa of the muscle, which correlates directly with muscle hypertrophy caused by exercise.^4^^,^^7^

## OBJECTIVE

The aim of this blinded randomized controlled trial was to evaluate the efficacy of pelvic floor muscle training and hypopressive exercises for increasing the CSA of the levator ani muscle in women with pelvic organ prolapse.

## METHODS

This was a single-blind randomized controlled trial. All participants received a three-month intervention according to their group allocation after the first evaluation, and received a second evaluation after the intervention period. This study was registered at Clinicaltrials.gov (NCT 01196598). This study was also approved by our institution’s Medical Ethics Committee, under reference number 1964/07. 

Between January 2009 and January 2010, 58 women with pelvic organ prolapse who were patients at the Urogynecology and Vaginal Surgery outpatient clinic of Universidade Federal de São Paulo were evaluated by a gynecologist during a routine consultation and were asked to participate. Those who agreed were included, and they provided written informed consent for their participation. Once enrolled, the subjects were stratified into three different groups: two treatment groups, with 21 patients each, consisting of PFM training (Group I [GI]), hypopressive exercises plus voluntary pelvic floor muscle contraction (Group II [GII]) and the control group (Group III [GIII]), with 16 patients. The group allocations were undertaken using computer-generated random numbers to stratify the randomization. The main investigation was blind to the group allocation. 

The inclusion criteria were the presence of stage II pelvic organ prolapse and not undergoing surgery to correct it during the study. Women with neuromuscular diseases and those undergoing hormone therapy were excluded.

The groups were homogeneous regarding age (P = 0.126), number of pregnancies (P = 0.353), number of vaginal deliveries (P = 0.106), body mass index (BMI) (P = 0.332) and menopausal status (P = 0.875) (Table 1).

During the clinical evaluation, pelvic organ prolapse (POP) was measured according to the POP-Q classification.^14^ We then evaluated the CSA of the levator ani muscle using two-dimensional transperineal ultrasonography.

**Figure 1.**
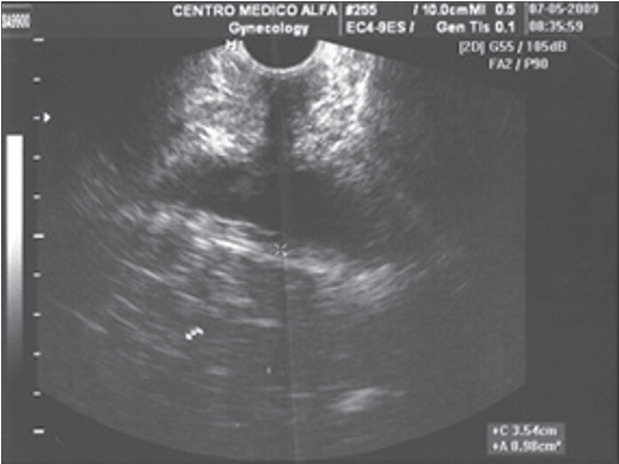
Ultrasound image of cross-sectional area of levator ani muscle.

The image obtained showed the length of the urethra and bladder neck. The levator ani muscle was viewed as a hypoechogenic structure bilateral to the urethra in the transverse plane (Figure 1). Subsequently, we calculated the CSA (in cm^2^). The value considered for analysis was the average of three measurements performed by one examiner on the left side. Our research group previously demonstrated that this examination had good reproducibility and interobserver correlation.^7^

After this evaluation, the subjects were stratified into the three groups described above. The women in GI and GII completed three initial training sessions, with different content for each group. The women in GI were taught how to properly contract the PFM alone. The women in GII were taught to contract the PFM in conjunction with hypopressive exercises, using diaphragmatic breathing, as recommended by Caufriez and Ballester.^15^ Both groups received home exercise programs as described below, with twice-monthly physiotherapy visits.

The daily home exercise protocol for GI included three sets of 8-12 close-to-maximal contractions per day, in lying down, sitting up and standing positions. Each contraction was held for 6-8 seconds.

The protocol for GII consisted of 10 repetitions of hypopressive exercises in lying down and standing positions, in association with PFM contractions for 3 to 8 seconds. Although the number of contractions differed between the two groups, the times spent on daily exercises were similar. The hypopressive exercises took a longer time to perform.

GIII had a single consultation with a physiotherapist, and received instructions to contract the pelvic floor muscles during increases in abdominal pressure, without following a defined protocol. 

The three groups received standardized lifestyle advice containing instructions on seeking advice, where appropriate, about weight loss, constipation, coughing and the avoidance of heavy lifting. 

The statistical analysis was performed using the Statistical Package for the Social Sciences (SPSS) 17.0. The power calculation for the study was based on the power estimations and results from previous studies that were designed to detect differences between groups^6^^,^^7^ with a significance level of 0.05. In these previous studies, significant differences in the same outcomes were shown for groups of 19 to 24 subjects. To estimate a difference between the two groups of 0.5 cm^2^ in the CSA of the levator ani muscle, with an alpha (type I) error of 5%, a power of 90% and a beta (type II) error of 10%, a sample of 15 subjects would be considered appropriate. The statistical significance of differences in CSA between different groups was assessed using nonparametric tests (Kruskal-Wallis). The ANOVA test, chi-square test and Mann-Whitney test were also used. P < 0.05 was considered statistically significant.

**Table 1.** Distribution of the patients according to demographic characteristics before the beginning of treatment

	Group I	Group II	Group III	P value
Age (years)	51.9 (± 7.4)	56.7 (± 10.7)	58.7 (± 10.4)	0.126*
Body mass index (BMI, kg/m2)	29.9 (± 3.5)	28.8 (± 3.9)	29.7 (± 2.7)	0.332*
Number of pregnancies	3.2 (± 2.2)	3.8 (± 1.8)	3.7 (± 3.2)	0.353*
Number of deliveries	1.8 (± 1.6)	2.4 (± 1.6)	3 (± 2.4)	0.106*
Menopausal status	15 (71%)	16 (76%)	11 (68%)	0.875†

*P value obtained using mann-whitney test; †p value obtained using chi-square test; α = 5%

## RESULTS

Sixty-three patients were included, i.e. 21 in each group. Among these, five patients in the control group were lost from the three-month revaluation: three of them dropped out, one moved away and one underwent corrective surgery for pelvic organ prolapse. Thus, 58 patients remained at the end of the study period. In groups GI, GII and GIII before treatment, the average CSAs were 1.65, 1.43 and 1.55 cm^2^, respectively (P = 0.130), as shown in Figure 1. After three months, there were significant differences in the CSAs: in GI, the CSA went from 1.6 (± 0.4) to 2.1 (± 0.3) cm^2^ (P < 0.001); and in GII, the CSA went from 1.4 (± 0.3) to 1.8 (± 0.5) cm^2^ (P = 0.001). The change in GIII was not statistically significant, from 1.5 (± 0.3) to 1.4 (± 0.3) cm^2^ (P = 0.816). These results are shown in Table 2.

**Table 2.** Correlation between ultrasound imaging for initial and final groups I, II and III

	Cross-sectional area	n	Mean (± SD)	P value
Group I	1^st^ examination	21	1.6 (0.4)	< 0.001^*^
	2^nd^ examination	21	2.1 (0.3)	
Group II	1^st^ examination	21	1.4 (0.3)	0.001^*^
	2^nd^ examination	21	1.8 (0.5)	
Group III	1^st^ examination	16	1.5 (0.3)	0.816
	2^nd^ examination	16	1.4 (0.3)	

SD = standard deviation; ^*^Analysis of variance test

Comparison between the groups showed that the results for GI and GII were similar (P = 0.078); GI and GIII were different (P < 0.001); and GII and GIII were also different (P = 0.015). This indicated that CSA after physiotherapy increased similarly in the two treatment groups.

## DISCUSSION

This study demonstrated that both PFM training and PFM training with transversus abdominis activation increase the CSA of the levator ani muscle, as assessed by two-dimensional ultrasonography. According to Ahtiainen et al., muscle CSA, as measured by ultrasonography, is associated directly with hypertrophy.^16^ A strong correlation between CSA and muscle strength or contractile capacity has also been shown.^17^ Therefore, this measurement directly reflects hypertrophy, and ultrasound is a valid and reproducible method for this measurement.^7^

In this study, we evaluated 58 women with pelvic organ prolapse before and after physiotherapy treatment. Two important factors directly influenced the initial CSA of the levator ani muscle of these women: age and the presence of pelvic floor dysfunction.

Women experience a substantial decrease in muscle mass (10% to 16%) between 50 and 60 years of age.^18^ There is also a decrease in CSA, infiltration of fat and connective tissue in muscle, a decrease in the size and number of muscle fibers and a decrease in the number of motor units.^18^ Considering the ages of the patients included in this study, we can infer that the levator ani muscles of these women had become weak and atrophied. The hypoestrogenic state of the women in this study added to the effects of age. As Jármy-Di Bella et al. demonstrated, estrogen deprivation weakens the thenar muscles and vessels of the pelvic floor and impairs their function, thereby leading to urinary incontinence and other dysfunctions.^19^

The presence of pelvic floor dysfunction is an aggravating factor for muscle atrophy. Hoyte et al. performed a cross-sectional study that assessed the volume of the levator ani muscle through magnetic resonance imaging. Thirty patients were included, divided into three groups: 10 asymptomatic patients, 10 with urinary incontinence and 10 with pelvic organ prolapse. The muscle volume values were 32.2 cm^3^, 23.3 cm^3^ and 18.4 cm^3^, respectively (P < 0.05). Note that the volume of the pelvic floor muscle was lower in women with pelvic organ prolapse.^12^ These data support our results, which showed a small CSA for the levator ani muscle in study subjects with pelvic organ prolapse. 

Oliveira et al. evaluated 63 premenopausal women divided into three groups: nulliparous, continent multiparous and incontinent multiparous. The CSA values were 1.83 cm^2^, 1.78 cm^2^ and 1.32 cm^2^, respectively.^7^ These results are similar to those of the present study, in which 58 women with pelvic floor dysfunction showed CSA of 1.53 cm^2^.

Through ultrasound evaluation, Bernstein observed that the CSA of the PFM decreased with age. Bernstein found that the muscles were thinner in women over 60 years of age than in younger women. There was also a negative correlation between age and strength of muscular contraction of the pelvic floor. Women with disorders have significantly thinner PFMs than do healthy women, but Bernstein stated that this difference could be eliminated through PFM training. These statements are concordant with the results from the present study: we found increased muscle volume in both training groups, compared with the control group (P < 0.001).^1^

Our study examined two muscle-training techniques: pfm training, which is the gold standard for treating stress urinary incontinence,^20^^,^^21^ and pfm training with transversus abdominis muscle activation through hypopressive exercises,^15^ both carried out daily for 12 weeks. There were significant increases in the csa of the levator ani muscle in both treatment groups, compared with the control group (p < 0.001). However, the group that underwent specific training (gi) improved by about 50%, while the hypopressive exercise group (gii) improved by about 20% after treatment. These values are in agreement with fleck and kraemer, who reported that the muscle csa increase ranged from 20% to 40% with a minimum of eight weeks of training.^22^

Bø and Aschehoug reported that exercises needed to target the specific muscle group to have positive results. The muscle behavior in the women in the group with specific training (GI) supports this statement, and this may explain this group’s greater increase in CSA, although the changes in both groups were statistically significant.^23^

Braekken et al. performed three-dimensional ultrasound scans on the levator ani muscle, the hiatus area and the resting position of the bladder and rectum in 109 women. The subjects had stage I, II or III pelvic organ prolapse and were randomized to either a treatment group or a control group. The treatment group performed PFM training exercises for six months under the guidance of a physiotherapist. The results showed a significant increase in muscle volume (an average of 1.9 mm; P < 0.05), a decrease in the genital hiatus area (P < 0.05), and an elevated position of the bladder and rectum featuring reduced pelvic organ prolapse (P < 0.05).^4^ These results are in accordance with the present study, which demonstrated increased muscle size, and therefore reduced pelvic organ prolapse, using two-dimensional ultrasonography.

Importantly, the examinations used for the present study were conducted by the same examiner, both before and after the treatment. Two-dimensional transperineal ultrasonography was chosen for CSA measurements on the levator ani because it is relatively inexpensive and does not cause discomfort to the patient. According to Dietz and Shek, translabial or transperineal arrangements provide direct access to the pelvic floor.^3^ Oliveira et al. demonstrated the validity and interobserver reproducibility of the technique used in the present study.^7^

More longitudinal studies with larger samples and longer follow-up are needed to understand the effect of PFM training on pelvic organ prolapse.

## CONCLUSIONS

The CSA of the levator ani muscle increases significantly with physiotherapy in women with pelvic organ prolapse. Pelvic floor muscle training and hypopressive exercises seem to produce similar improvements in the CSA of the levator ani muscle.
